# Psychological Flexibility and Self-Compassion as Predictors of Well-Being: Mediating Role of a Balanced Time Perspective

**DOI:** 10.3389/fpsyg.2021.671746

**Published:** 2021-06-10

**Authors:** Anna Pyszkowska, Michael Rönnlund

**Affiliations:** ^1^Institute of Psychology, University of Silesia in Katowice, Katowice, Poland; ^2^Department of Psychology, Umeå University, Umeå, Sweden

**Keywords:** psychological flexibility, self-compassion, time perspective, DBTP, well-being

## Abstract

Measures of psychological flexibility and self-compassion are strongly associated with well-being. The aim of the present study was to test the hypothesis that these relationships are mediated by a balanced time perspective, a proposed ideal way of relating to the past, present, and future that may correspond with an ability to flexibly switch temporal focus. For this purpose, a Polish community sample (*N* = 421) responded to a web-survey including measures of psychological flexibility (AAQ-II), self-compassion (SCS), two measures of positive aspects of well-being (Satisfaction with Life, Quality of Life), and the Zimbardo Time Perspective Inventory (ZTPI). Structural equation models, involving a measure of deviation from a balanced time perspective (DBTP) as a mediator of relationships between latent-level psychological flexibility, self-compassion and well-being factors, were tested. We examined separate models for psychological flexibility and self-compassion and a model including both constructs. The results for separate models were consistent with partial mediation of relationships with well-being, both for psychological flexibility and self-compassion. Results for the analysis involving both constructs, suggested unique contributions of both to DBTP, which in turn predicted well-being, but the link between psychological flexibility and DBTP appeared to be the strongest. In further analyses, three ZTPI dimensions were identified as most critical, namely Past Positive, Present Fatalistic, and Past Negative, each of which were part of an indirect effect on well-being. Psychological flexibility in particular, showed a strong negative association with a Past Negative orientation. Taken together, the results indicate that time perspective is a factor to understand the links between psychological flexibility/self-compassion and well-being. While the results pertaining to self-compassion were consistent with results of a couple of prior studies, this is, to our knowledge, the first demonstration of a link between psychological flexibility and a balanced time perspective. These findings should be relevant for clinical research and practice.

## Introduction

Third wave cognitive and behavioral therapies (e.g., Acceptance and Commitment Therapy, or ACT and Compassion-Focused Therapy) share the core aim of helping people to become aware of their thoughts and treat them in a non-judgmental way (Hayes et al., 1999). These therapies and underlying theoretical frameworks introduced several psychological constructs important to understand differences in mental health. In the present study two such constructs, namely psychological flexibility (Hayes et al., 1999) and self-compassion (Neff, 2003), were examined as predictors of positive aspects of well-being. Additionally, we considered a construct derived from another research tradition, namely a balanced time perspective (Zimbardo and Boyd, 2008) as a potential medium through which psychological flexibility and self-compassion influence well-being.

Psychological flexibility has been defined as the ability to stay in contact with the present moment, regardless of unpleasant thoughts, feelings, and sensations, while choosing and developing one’s behavior repertoire based on personal values and situational contexts (Hayes et al., 1999). In other words, own thoughts and emotions are held a bit more lightly, and long-term values and goals are acted on, rather than current thoughts, impulses and feelings, in a flexible manner. Four elements beyond those concerned with values and committed actions are proposed: mindfulness, i.e., being in contact with the present moment, acceptance, i.e., allowance of inner experience in a non-judgmental way, defusion, i.e., detachment of thoughts, and, finally, self as context, which is realizing that you are experiencing thoughts rather being the content of these thoughts. Instruments used to assess psychological flexibility include version of the Acceptance and Action Questionnaire-II (AAQ-II, Bond et al., 2011).

In the ACT framework (Hayes et al., 1999) psychological flexibility is regarded as a fundamental aspect of psychological health. In line with its core assumption, measures of flexibility were significant predictors of a number of mental health indices, for example, measures of anxiety, depression, alexithymia, and work performance (for a review, Kashdan and Rottenberg, 2010). As concerns positive aspects of well-being, a growing number of studies involving clinical (McAteer and Gillanders, 2019) and non-clinical samples (Steenhaut et al., 2018) additionally demonstrate that psychological flexibility is associated with positive aspects of well-being. Importantly, interventions designed to develop psychological flexibility also reduced symptoms of psychological distress and increased quality of life ratings (Meyer et al., 2018).

Self-compassion, in turn, is understood as being warm and understanding toward own suffering, failure, and feelings of inadequacy when they arise (Neff, 2003). Neff proposed three basic components. Self-kindness, concerns acceptance of one’s own difficulties rather than ignoring them or being harshly self-critic. Common humanity is realizing that suffering and personal inadequacies are part of a shared human experience and that one is not alone in aspects of imperfection or suffering. Mindfulness, finally, involves a non-judgmental, receptive mind state where thoughts and feelings related to own suffering are perceived as they are rather than being over-identified with.

Evidence in support of the notion that self-compassion is a major health factor includes significant negative associations of self-compassion as a trait with measures of perceived stress (Neely et al., 2009; Eriksson et al., 2018), symptoms of depression (Neff, 2003; Raes, 2010), anxiety (Neff, 2003), and burnout (Eriksson et al., 2018). Higher levels of self-compassion were, by contrast, linked to positive aspects of well-being, for example greater satisfaction with life (Neff, 2003; Neely et al., 2009) and professional quality of life (Beaumont et al., 2016). Apart from demonstrations of associations between measures of self-compassion and measures of mental health, interventions aimed to cultivate self-compassion showed that the resulting gains in self-compassion were accompanied by improvements across multiple aspects of well-being (Neff and Germer, 2013).

Taken together, there is much evidence psychological flexibility and self-compassion are critical to aspects of well-being. Given a similar association with various outcome variables, and the shared feature in form of mindful awareness of unpleasant emotions, it seems relevant to consider the covariation of measures of the two constructs and the extent to which they account for unique variance in the prediction of well-being. Results from a few studies in which measures of both were administered (Marshall and Brockman, 2016; McLean et al., 2018; Pyszkowska, 2020) confirm that they are significantly correlated, but, still, the magnitude of the association (weighted average of around *r* = 0.50 across studies) indicates much non-shared variance. Importantly, studies by Woodruff et al. (2014) and by Marshall and Brockman (2016) suggest that psychological flexibility and self-compassion account for a significant amount of unique variance in the prediction of life satisfaction. One thing that needs to be clarified are the paths or mechanisms through which psychological flexibility and self-compassion support positive aspects of well-being, beyond a mere reduction of distress. Influencing people’s time perspective, we argue, could be one factor.

Lewin (1951) originally proposed that an individual’s mood, behavior and morale depends on their psychological view of the past and future that exists a given time. Building on these ideas, Zimbardo and Boyd (1999) conceptualized time perspective as attitudes, beliefs and values of the individual in relation to the past, present and future. These are, they hypothesized often used automatically, develop early and once established, habitual and trait-like in nature. These beliefs and attitudes, in turn, are critical to the way one interprets and experiences events, and, consequently, one’s reaction to them. The most widely used metric to capture individual differences in time perspective is Zimbardo Time Perspective inventory (ZTPI, Zimbardo and Boyd, 1999). ZTPI distinguishes five dimensions. Two dimensions concern the past, two capture the present, and one concerns the future. Past Positive involves a warm and nostalgic relation to the personal past, whereas Past Negative captures an aversive view of the past. Present Hedonistic captures a live-for-the-moment attitude that involves instant gratification and little concern for future consequences of behavior. Fatalistic involves a helpless view of the present characterized by external rather than internal locus of control. Future, finally, captures attitudes and behavioral intentions characterized by striving for future rewards. A basic tenet in the framework underlying ZTPI is that biases, in the form of overuse of one, or several, of the dimensions (time perspective biases), may predispose the individual to mental health problems and problematic behaviors. A wealth of studies are in line with this prediction, and specific time perspective biases were are, for example, linked to stress (Papastamatelou et al., 2015; Rönnlund et al., 2018), anxiety (Åström et al., 2018), and depression (Desmyter and De Raedt, 2012; Åström et al., 2019).

Apart from the separate ZTPI dimensions, a recent research focus has been on the totality of an individual’s temporal focus and attitude. This research was based on the notion of a balanced time perspective (BTP). Zimbardo and Boyd (1999) originally described BTP as involving the ability to mentally switch between temporal orientations in adaptive way, something which may serve to avoid getting stuck in some particular temporal frame or attitude, for example an overly negative past perspective.

The most widely used and validated method to capture BTP is a measure referred to as deviation from a balanced time perspective (DBTP, Stolarski et al., 2011; Zhang et al., 2013; for a review of research, Stolarski et al., 2020). DBTP reflects the degree to which an individual’s entire ZTPI score profile deviates from a proposed optimal one (Zimbardo and Boyd, 2008), characterized by a high score on the Past Positive dimension, a moderately high score on Present Hedonistic and Future and a low score on the Past Negative and Present Fatalistic dimensions.

As such, the conceptualization of BTP as an ability to switch temporally, and thereby adapt to an ever-changing environment, shows obvious similarity with the conceptualization of psychological flexibility In fact, being able to flexibly switch between temporal perspectives, may be considered as a specific expression of psychological flexibility (Kashdan and Rottenberg, 2010). Despite the similarity in terms of conceptualization of constructs, we could not find any study that examined associations between a measure of psychological flexibility and indicators of BTP (e.g., DBTP). With regard to one the presumed subcomponents of flexibility, namely mindfulness, several studies indicate a substantial relationship with DBTP, though, such that higher levels of trait mindfulness are associated with a smaller DBTP (Stolarski et al., 2016; Rönnlund et al., 2019). Moreover, data were consistent with models involving DBPT as a mediator of the relationship between mindfulness and satisfaction with life (Stolarski et al., 2016) and between mindfulness and perceived stress (Rönnlund et al., 2019), which as noted was linked to psychological flexibility. As concerns self-compassion, two studies indicated a significant association between self-compassion and DBTP (Philips, 2018; Ge et al., 2019), higher levels of self-compassion being predictive of smaller deviations from BTP. In addition, the results by Philips (2018) indicated that DBTP mediated part of the relationship between self-compassion and a measure of life satisfaction.

Turning to separate ZTPI time dimensions, those showing the strongest association with life satisfaction and related measures are typically Past Negative and Past Positive (Desmyter and De Raedt, 2012; Muro et al., 2017). With regard to the relation to the past (or future), it should be considered that evaluations and interpretations of are made from viewpoint of the present (Zimbardo and Boyd, 2008; cf. presentification, Mead, 1932). This leaves room for resources supporting more adaptive ways of coping with adverse events in the past (or fears of future events) to operate. When it comes to processing and interpretation of past events, several findings appear to be consistent with the idea that self-compassion can reduce past negativity. Miyagawa and Taniguchi (2020) reasoned that self-compassion might facilitate a “high-level construal” that serves to increase subjective distance to negative events by virtue taking a balanced view of own suffering and understanding of aspects of the event as part of the human condition, rather than merely a personal problem. A low-level construal, involving a more concrete understanding and no little decontexutalization of the target features of an event, would, by contrast, be more likely be used when self-compassion is lacking, making the negative events appear close. In line with these predictions, participants’ ratings of the past negative events differed depending on level of self-compassion: Self-compassion was associated with greater perceived distance from negative events, and the data were consistent with a model by which this perception of distance mediated the relation between self-compassion and negative mood reactions.

Another means by which self-compassion might alter perception of the past and improve well-being is by reducing negatively toned repetitive thinking in form of rumination. Rumination tends to be directed toward the past (Nolen-Hoeksema et al., 2008) and, as demonstrated by Åström et al. (2018), positively associated with scores on the ZTPI Past Negative dimension. Consistent with the hypothesis that self-compassion may block rumination, Odou and Brinker (2015) demonstrated short-term benefits of a simple self-compassionate writing task to approach an induced negative mood state, with greater effect for individuals identified as high ruminators. In a related vein, Samaie and Farahani (2011) found that high levels of self-compassion attenuated the association between measures of rumination and stress. Rumination is sometimes, by itself, taken as an instance of cognitive inflexibility and psychological inflexibility as measured by the AAQ-II was associated with a measure of rumination (Cernvall et al., 2015).

In summary, psychological flexibility seems conceptually linked to the notion of a BTP, but empirical evidence in form of associations between measures of the constructs is, to our knowledge, lacking. Self-compassion was found to be correlated with a measure of BTP (DBTP), which in turn is a well-established predictor of well-being. Additionally, a core element of both constructs, mindfulness, was found to be associated with lower DBTP. In addition, there are several indications that psychological flexibility and self-compassion could serve to improve temporal harmony as far as more specific dimensions of time perspective are considered, for example by reducing the influence of past negative events.

The objective of the present study was to examine association of psychological flexibility, self-compassion, time perspective, and positive aspects of well-being, with a particular focus on the hypothesis that relationships between each of the first two constructs and well-being are mediated by DBTP. To this end, separate models for psychological flexibility and self-compassion as well as models involving both as predictors of well-being were tested. As variations in DBTP could reflect a variety of different time perspective biases, corresponding analyses were in addition performed at the level of separate ZTPI dimensions (Rönnlund et al., 2017; 2019), i.e., to determine whether some particular facets of time perspective are critical to the expected links between psychological flexibility/self-compassion and well-being.

## Materials and Methods

### Participants

A community sample including 431 individuals (including 230 women, *M*_*age*_ = 28.97, *SD*_*age*_ = 12.04) was recruited using snowball sampling from the general population of the inhabitants of Poland via an Internet survey. As regards educational background, 57.1% of the respondents reported having completed secondary education, 35.5% higher education, and 7.4% elementary education. The survey was anonymous and voluntary. Informed consent was obtained from all participants.

### Instruments

#### Psychological Flexibility

The Acceptance and Action Questionnaire-II developed by Bond et al. (2011) in the Polish adaptation by Kleszcz et al. (2018) was used as a measure of psychological flexibility. Flexibility is measured using seven statements (e.g., “I’m afraid of my feelings”) rated on a Likert scale from 1 (always untrue) to 7 (always true). The higher the score obtained by the respondent, the lower the level of psychological flexibility, but for the purpose of this study a reversed sum score was used, i.e., such that in the present study higher scores equated to greater psychological flexibility. Cronbach’s α was α = 0.90.

#### Self-Compassion

An abridged version of the Self-Compassion Scale (SCS), developed by Raes et al. (2011) and translated to Polish (by Kocur, unpublished) was used as a measure of self-compassion. This version includes 12 statements (e.g., “I try to see my failings as part of the human condition”) that are rated on a scale from 1 (does not describe me at all) to 5 (describes me very well). In common with the full version of the SCS (Neff, 2003) items reflect common humanity (two items) vs. Isolation (two items), Mindfulness (two items) vs. Over-identification (two items) and self-kindness (two items) vs. Judgment (two items). Cronbach’s α for entire Scale was α = 0.74.

#### Time Perspective

The Polish adaptation of the Zimbardo Time Perspective Inventory (Zimbardo and Boyd, 1999) in Polish translation by Cybulko and Zieliński was used to assess time perspective. The questionnaire consists of 56 statements concerning time (e.g., “Happy memories of good times spring readily to mind”) rated on a 5-point Likert scale ranging from very untrue (coded as 1) to very true (coded as 5). The average rating of items assigned to each of five dimensions are considered: Past Negative (α = 0.84, in the present sample), Present Hedonistic (α = 0.82), Future (α = 0.79), Past Positive (α = 0.66), and Present Fatalistic (α = 0.68).

Deviation from a balanced time perspective was calculated in accord with the formula in Stolarski et al. (2011):

$$\sqrt{{\left(\text{oPN}-\text{ePN}\right)}^{2}+{\left(\text{oPP}-\text{ePP}\right)}^{2}+{\left(\text{oPF}-\text{ePF}\right)}^{2}}$$

$$+{\left(\text{oPH}-\text{ePH}\right)}^{2}+{\left(\text{oF}-\text{eF}\right)}^{2}$$

Where o = optimal score and e = empirical (i.e., observed) score. In line with Stolarski et al. (2011), optimal scores were set to: oPN = 1.95, oPP = 4.6, oPF = 1.5, oPH = 3.9, and oF = 4.0.

#### Well-Being

To capture multiple aspects of well-being two questionnaires were included. The first was the Quality of Life Questionnaire by Straś-Romanowska (2005), referring to the multidimensional human concept. This instrument consists of 60 statements (“I feel that I have found my place in life”) and defines four dimensions of quality of life: psychophysical (biological, α = 0.74), psychosocial (social relationships and social identity, α = 0.80), subjective (sense of autonomy, α = 0.78) and metaphysical (spiritual, α = 0.75). For the purposes of the present study the total score (α = 0.90) was used as an indicator of well-being. The second instrument was the Satisfaction with Life Scale (SWLS) by Diener et al. (1985), in the Polish adaptation by Juczyński (2001). SWLS consists of five statements (e.g., “In most ways my life is close to my ideal”) rated on a Likert scale from strongly disagree (1) to strongly agree (7), and the sum of rating across items reflects the global sense of satisfaction with life. Cronbach’s α was α = 0.86.

### Statistical Methods

Structural equation modeling (SEM) procedures were employed using IBM SPSS AMOS 26. In the SEM-models, a parceling approach procedure was used to reduce the number of observed variables in the model. This results in a more parsimonious model while providing increased power to test the relations among latent variables (Little et al., 2013). In accord with several prior studies (Joeng and Turner, 2015; Yang et al., 2019) three indicators were created on the basis of items from the abridged version of the Self-Compassion Scale by computing the average score for self-kindness and the reverse scored self-judgment (SC1), common humanity and the reverse scored isolation (SC2), and mindfulness and the reverse scored over-identification (SC3). Psychological flexibility as reflected by AAQ II is a unitary construct (Bond et al., 2011; Costa et al., 2014): Hence three item parcels (PF1 for items 1, 3,6; PF2 for items 4,7; and PF3 for items 2, 3) were created using random assignment (Matsunaga, 2008). Finally, a latent well-being factor reflected two measures (Quality of life and Satisfaction with Life). Model fit was evaluated using the following indexes: Comparative Fit Index (CFI), were values >0.95 are considered to indicate good model fit, and Squared Error of Approximation, RMSEA, for which values <0.06 are considered to indicate good model fit and values of 0.08 or lower are often taken to indicate reasonable model fit (Hu and Bentler, 1999). In line with prior studies, we additionally report χ^2^ statistics, even though it is widely recognized that this is not a good index of model fit due to being heavily dependent on sample size. To test the significance of indirect effects, we used a bootstrap procedure involving 3,000 bootstrap samples and computation of 95% bias-corrected confidence intervals (BCIs) of the estimates.

## Results

### Descriptive Statistics

Means, standard deviations, indicator of distribution form (skewness and kurtosis) for the major study measures, are presented in Table 1, together with zero-order correlations. The means appear to be comparable with those in prior studies involving non-clinical samples, with no values for skewness or kurtosis approaching cutoff values (e.g., 1 and 2, respectively). The measures of psychological flexibility and self-compassion showed a positive association near the border from medium to large (*r* = 0.53). The correlational data furthermore confirmed positive associations between psychological flexibility and self-compassion on the one hand and the measures of well-being on the other. In turn, psychological flexibility and self-compassion were negatively associated with DBTP. Finally, DBTP was negatively associated with both measures of well-being (Quality of Life, Satisfaction with Life Scale).

**TABLE 1:** Descriptive statistics and zero-order correlations of study measures.

**Measure**	***M***	***SD***	**Skewness**	**Kurtosis**	**1**	**2**	**3**	**4**
(1) Psychological flexibility^a^	21.33	9.15	0.64	–0.05	–			
(2) Self-compassion	35.77	7.69	–0.20	–0.33	0.55***	–		
(3) DBTP	2.42	0.73	0.42	0.17	−0.50***	−0.44***	–	
(4) Quality of life	182.8	19.42	–0.33	–0.09	0.52***	0.51***	−0.61***	–
(5) SWLS	21.17	6.00	–0.28	–0.22	0.49***	0.45***	−0.51***	0.66***

^*a*^Reverse scoring of AAQ II total.

***p < 0.001.

### Mediation Models Involving DBTP

We first tested a measurement model involving all relevant constructs, i.e., psychological flexibility (reflected by three indicators), self-compassion (three indicators), DBTP (manifest variable), and well-being (two indicators) as correlated factors. All item loadings were high (βs > 0.60) and values for fit indexes, CFI = 0.993, RMSEA = 0.037 (90% CI 0.009–0.059), χ^2^ (22) = 35.0. *p* = 0.04, indicated good model fit.

**FIGURE 1:**
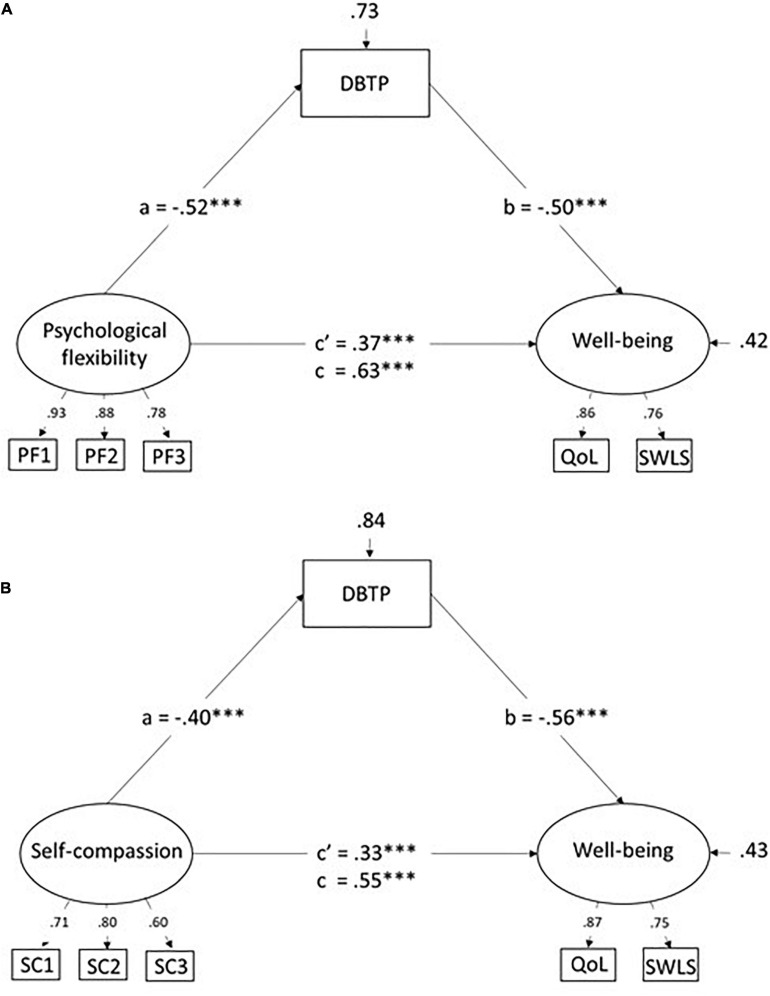
**(A,B)** Models involving deviations from a balanced time perspective (DBTP) as a mediator of the relationship between psychological flexibility and well-being **(A)** or between self-compassion and well-being **(B)**. Values are standardized coefficents. c’ = direct effect, c = total effect.

Next, structural models tested. These included psychological flexibility and self-compassion factors as the predictor(s) of well-being, and DBTP as a mediator of this relationship. Separate models - one for psychological flexibility and one for self-compassion–were first evaluated. These models are depicted in Figure 1A (psychological flexibility) and 1B (self-compassion) together with standardized regression weights for direct effects, and residual variance for the endogenous variables (DBTP and well-being).

Each model showed good fit; CFI = 0.993, RMSEA = 0.058, χ^2^(7) = 17.1, *p* = 0.02 for the model in a) and CFI = 1.00, RMSEA = 0.008, χ^2^(7) = 7.2, *p* = 0.41, for the model in b). Beginning with the model involving psychological flexibility (1a), a significant negative association from psychological flexibility to DBTP consistent with predictions was observed. The value for residual variance indicates that 27% (1-0.73) of the variance in DBTP was accounted for by psychological flexibility. In turn, DBTP showed a significant negative association with well-being and the indirect effect (psychological flexibility to well-being via DBTP) being was significant (*p* < 0.001) as judged by result from the bootstrap analysis. Together, 58% of the variance well-being was accounted for by the variables. The direct effect (c’) of psychological flexibility on well-being was positive and significant. Still, as indicated by 95% BCIs, the value for c’ (0.26–0.49) was significantly lower than the total effect (*c* = 0.63), meaning, a second criterion of (partial) mediation, apart from the significant indirect effect, was met.

Patterns of results for the model that included self-compassion (Figure 1B) were highly similar to those for the first model. More specifically, a significant negative association between self-compassion and DBT was evident (*R*^2^ = 0.16, i.e., somewhat smaller than for psychological flexibility). In turn, DBTP showed a negative association with well-being (β = −0.56). Furthermore, bootstrap analyses indicated a significant indirect effect on well-being via DBTP (β = 0.22, *p* < 0.001) and a significant difference between c’ and c. Thus, as for psychological flexibility, the results were consistent with the hypothesis that DBTP mediates part of the relationship with well-being.

**FIGURE 2:**
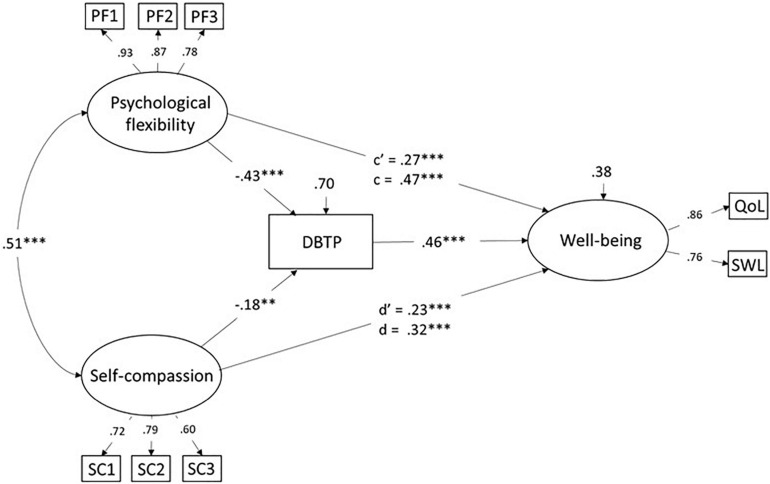
Structural model involving DBTP as a mediator of the relationship between psychological flexibility, self-compassion, and well-being. Values are standardized coefficents. c’ = direct effect, c = total effect.

Given results consistent with a role of DBTP as mediator of relationships with well-being for psychological flexibility as well as self-compassion, we tested a corresponding model (i.e., with DBTP as a mediating variable) including both factors as the predictors. As before, model fit for this model was good, judged by values for fit indexes, CFI = 0.993; RMSEA = 0.037, χ^2^(22) = 35.0, *p* = 0.04. The model is presented in Figure 2.

The estimate of association of psychological flexibility and self-compassion as latent factors (*r* = 0.51) was close to that for manifest sum scores (cf. Table 1). Psychological flexibility, self-compassion, and DBTP together accounted for a substantial amount (63%) of the variance in well-being. This figure might be compared with the corresponding ones for the separate models (Figure 1) for an estimate of the additive (unique) contribution of psychological flexibility and self-compassion, respectively. The difference in *R*^2^ between models for separate or combined factor amounted to 5 or 6%.

Critically, paths from psychological flexibility (β = 0.43, 95% BCI -0.30 to -0.55), and from self-compassion (β = 0.18, 95% BCI -0.04 to -0.31) to DBTP, were both significant (*ps* < 0.01), even though as indicated by the BCIs, the former was the stronger predictor. As demonstrated before, DBTP was in turn negatively related to well-being (β = -46). The indirect effects, i.e., from psychological flexibility trough DBTP to well-being (β = 0.20, *p* < 0.001) and from self-compassion through DBTP to well-being (β = 0.07, *p* = 0.01) were both significant. Moreover, both for psychological flexibility (β = 0.27) and self-compassion (β = 0.23) significant direct effects on well-being were observed, i.e., associations independent of DBTP. A comparison of the values for the direct and total effect (i.e., c’ vs. c and d’ vs. d) suggests lower values for c’ and d’, though. The bootstrap analyses confirmed a statistically significant difference for psychological flexibility (95% BCI for c’ 0.16–0.42, <0.45 for c), but not for self-compassion (BCI for d’ 0.11–0.36, relative to *d* = 0.32).

### Multiple Mediation Models Involving Separate Time Perspective Dimensions

Provided findings consistent with the idea that DBTP, mediates part of the relationship between psychological flexibility and wellbeing, and consistent with an indirect link in the same direction, at least, for self-compassion, we turned to the issue of whether some temporal biases are of particular importance. For this purpose, multiple mediator models involving separate ZTPI dimensions (subscales) were tested. Some of the ZTPI dimensions are moderately associated (Zimbardo and Boyd, 1999). To restrict the number of variables in the model, only the dimensions that met the following criteria were considered: (a) showing a significant bivariate association with the welling factor, and (b) showing a significant association with one or both of the psychological flexibility and/or self-compassion factors. For this purpose, we considered a measurement model, corresponding to that tested initially, including the five ZTPI dimensions as correlated variables.

Except for Present Hedonistic (*r* = 0.07, *p* = 0.18), the dimension exhibited a significant (*p*s < 0.01) simple association with the well-being factor, positive in direction for Past Positive (*r* = 0.53) and Future (*r* = 0.29), and negative in direction for Past Negative (*r* = -0.57) and Present Fatalistic (*r* = -0.30). Three dimensions, in turn, showed a statistically significant association with psychological flexibility and/or self-compassion (*p*s < 0.01). These were Past Positive (*r* = 0.24 with psychological flexibility, *r* = 0.21 with self-compassion), Past Negative (*r* = -0.70 with psychological flexibility, *r* = -0.44 with self-compassion), and Present Fatalistic (*r* = -0.29 with psychological flexibility, *r* = -0.16 with self-compassion), while the Future dimension was not related to any of the two construct (*r* = -0.03 with psychological flexibility and *r* = 0.00 with self-compassion). Hence, Past Positive, Past Negative, and Present Fatalistic were included in the multiple mediator models.

**FIGURE 3:**
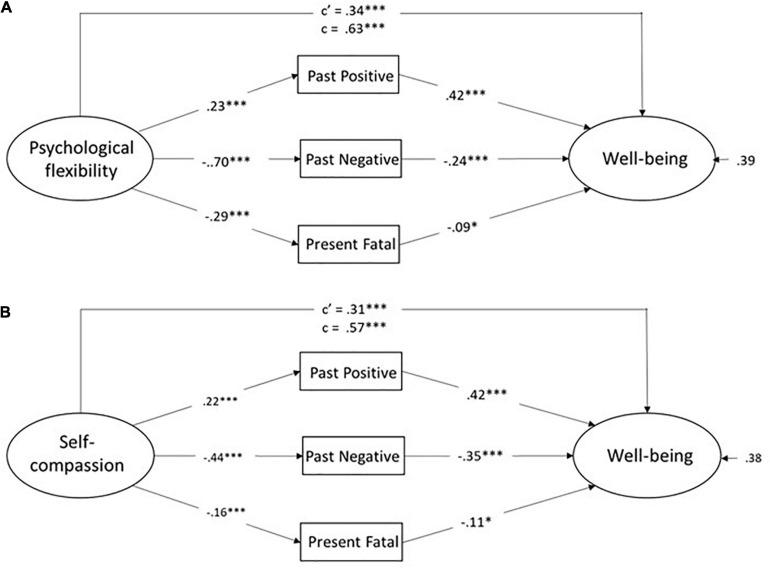
**(A,B)** Models involving ZTPI Past Positive, Past Negative, and Present Fatalistic as multiple mediators of the relationship between psychological flexibility and well-being **(A)** and between self-compassion and well-being **(B)**. Values are for standardized coefficents. c’ = direct effect, c = total effect.

Consistent with steps taken in analyses of DBTP, separate models for psychological flexibility and self-compassion first examined, followed by a model involving both. Given a medium positive association of Past Negative and Present Fatalistic in the current data set (*r* = 0.38) similar to that reported in prior studies, residual (error) correlated error terms were allowed for these two. The separate models for psychological flexibility and self-compassion as mediators are presented in Figures 3A,B.

The model values indicated good or acceptable model fit for both models (CFI = 0.99, RMSEA = 0.08, χ^2^(15) = 34.9, *p* < 0.01 for the first (3a) and CFI = 0.98, RMSEA = 0.06, χ^2^(15) = 37.8, *p* < 0.01, for the second (3b). A similar pattern was evident across in both models in that the endogenous factor was a predictor of each of the three ZTPI dimensions, i.e., higher Past Positive and lower scores on Past Negative and Present Fatalistic. In turn, each of the ZTPI dimension were significantly associated with well-being, with a positive association for Past Positive and a negative association for Past Negative and for Present Fatalistic. All three indirect effects (via each of the three ZTPI dimensions) were statistically significant in both models (*p*s < 0.05).

**FIGURE 4:**
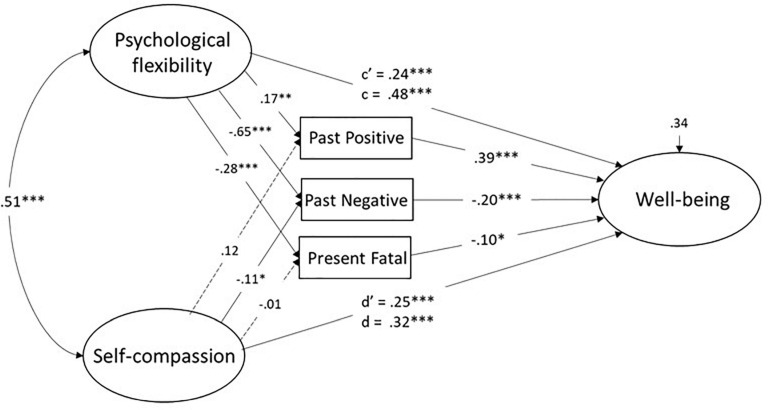
Structural model with hypothetized links between involving psychological flexibility, self compassion, and well-being with separate ZTPI dimensions as mediators. Values are for standardized coefficents. c’ = direct effect, c = total effect.

In a final step, we tested a multiple mediator model involving both psychological flexibility and self-compassion (cf. Model 2). The model is depicted in Figure 4 with estimates of associations of constructs and residual variance for the exogenous variables. Values for fit indexes were indicative of good model fit of the model, CFI = 0.98, RMSEA = 0.048, χ^2^(34) = 67.2, *p* < 0.01. The results pertaining to psychological flexibility replicated those in 3a to the extent that (a) significant links to the ZTPI dimensions were observed, and (b) each of three indirect effects were significant. For self-compassion, a significant unique association with Past Negative was observed, together with a significant indirect path to well-being via this dimension (*p* < 0.05).

## Discussion

The results confirmed patterns in prior studies but also provided some novel findings. In accord with other studies, psychological flexibility (Doorley et al., 2020) as well as self-compassion (Stoeber et al., 2020; Pandey et al., 2021) were strong predictors of well-being. Our results were also consistent with findings that each factor accounts for unique variance in well-being in prior (Woodruff et al., 2014; Marshall and Brockman, 2016). In addition, the results confirmed previous observations of a correlation of psychological flexibility and self-compassion of a magnitude around the boarder of medium to strong (slightly above *r* = 0.50) regardless of whether manifest sum scores for AAQ-II and SCS or latent-level factors are considered.

Of primary concern, the results demonstrated an association between each of the former constructs and the measure of BTP, higher levels of flexibility and self-compassion being associated with smaller DBTP value. Consistent with hypotheses underlying the mediation models, data indicated an indirect influence from each of the two factors on well-being through reductions in DBTP. Partial mediation was supported, meaning that other factors must be considered regardless of the validity of these links. With regard to self-compassion, the predicted chain of association involving DBTP as mediator is in agreement with results of two prior studies (Philips, 2018; Ge et al., 2019). By contrast, the finding that the presumed flexibility of shifting between temporal frames in an adaptive way, as reflected by a low DBTP value, was predicted by a broader psychological flexibility factor is, to our knowledge, a novel finding. In fact, psychological flexibility actually appeared to be the stronger predictor of DBTP as judged by the model involving self-compassion and psychological flexibility as predictors, even though self-compassion accounted for significant unique variance. The fact that a relatively small amount of increment in amount of variance accounted for was seen when both the flexibility and self-compassion factors were included, relative to one of these, seem to indicate what is common to both constructs, presumably a mindful and accepting view, is a major factor. As such, this would be consistent with previous demonstrations of a link between measures of mindfulness and DBTP both as reflected by cross-sectional associations (Stolarski et al., 2016; Rönnlund et al., 2019) and demonstrations that mindfulness-based interventions might serve to reduce DBTP (Rönnlund et al., 2019). Future studies might include mindfulness in addition to the construct examined here (cf. Woodruff et al., 2014) to evaluate this further.

DBTP is a summary measure reflecting biases over multiple ZTPI dimensions. This means that different individual may achieve the same deviation score based on rather different time perspective biases and we aimed to examine which were particularly important to the association of psychological flexibility, self-compassion, and well-being. The analyses suggested that three ZTPI dimension, namely Past Positive, Past Negative, and Present Fatalistic are critical. The observation that well-being was associated with higher scores on Past Positive and lower scores on the latter two dimensions is in line with patterns in other studies (Desmyter and De Raedt, 2012). Second, psychological flexibility and self-compassion were significant predictors of these three ZTPI dimension, even though, as for the analyses of DBTP, the results of model including both favored psychological flexibility. The finding of a positive association with Past Positive is interesting in the sense that self-compassion and psychological flexibility may not only reduces negatively valenced facets of time perspective but support a positive one. A reduction of a Present Fatalistic attitude seems consistent with elements of psychological flexibility. The association with Past Negative was the strongest in magnitude though, in particular for psychological flexibility for which a negative association with Past Negative was evident regardless of whether self-compassion was entered as a simultaneous predictor. This pattern is much in line with the concept of psychological rigidity and fusion, exemplified by ruminations (Åström et al., 2018), being stuck in the negative perception of the past (Ge et al., 2019) and the lack of acceptance of one’s suffering, including negative events in the past (Hayes et al., 1999). As there are no prior reports regarding relationships between psychological flexibility and time perspective to date, it can be only speculated that psychological inflexibility, characterized by strong fusion with one’s views and attitudes toward oneself and the world as well as small acceptance of one’s suffering can be strongly associated with negative thoughts and the feeling of “being stuck” in a negatively perceived past (cf. Harris, 2009; Åström et al., 2018).

By contrast, no evidence of a mediating role was obtained for the Future dimension. As such the finding of a relatively small association between ZTPI Future and well-being is consistent with several prior studies (Drake et al., 2008; Muro et al., 2017; Rönnlund et al., 2017). Given indications that flexibility and self-compassion promote healthier present and past TPs, one might expect these factors to promote a more positive future time perspective as well. In fact, results in Philips (2018) were taken to indicate that future outlook is improved by self-compassion and mediates the relationship between self-compassion and well-being. A difference in measures used to assess future perspective between studies is one factor to consider. In the study by Philips (2018), the future-related measure that mainly accounted for the link between self-compassion and well-being in a multiple mediator model was a measure of savoring-anticipating. Savoring-anticipation, involves deriving pleasure from anticipating future positive events. A measure of optimism, i.e., holding positive expectations about future experiences, was in addition reported to mediate (part of the) the relationship between self-compassion and well-being when each were entered in a single mediator model. While these measures of future outlook focus on one’s attitude toward the future in a more general terms and mainly concern valence, many ZTPI Future items reflect behavioral habits and cognitions indicative of planning or thinking ahead (time orientation). Thus, it might be that a positive future outlook in terms of valence/attitude are more relevant for the relationships with well-being. Finally, the unitary Future scale in the original version of the ZTPI that was used in the present study, has been regarded to miss an important aspect of future time perspective in the form of negative anticipations and worry of the future (Carelli et al., 2011). To overcome this limitation, a separate Future Negative dimension was developed (Carelli et al., 2011). Unlike Future, Future Negative shows a substantial (negative) association with well-being (Rönnlund et al., 2017) and mindfulness (Rönnlund et al., 2019). Significant associations between psychological flexibility/self-compassion and worry or anxiety (Kashdan and Rottenberg, 2010; Neff and Dahm, 2015) that relate to aspects of a future negative perspective, in addition seem to speak in favor of a potential association, although the hypothesis that psychological flexibility and self-compassion are significantly associated with future-negative facet of time perspective remains to be tested.

## Limitations and Future Research Directions

Despite strengths such as an adequate sample size, the present study has limitations. Of primary concern, the study design was cross-sectional and based on a heterogenous non-clinical convenience sample. It must be highlighted that longitudinal studies concerned with the development of self-compassion and psychological flexibility are required in order to provide an evaluation of causal relationships among the variables studied. Even though values for internal consistency for some of the ZTPI scales (e.g., Past Positive and Present Fatalistic) should probably be regarded as passable for research purpose they were a bit lower than in the original study by Zimbardo and Boyd (1999) and below the common cutoff value α > 0.70 (Nunnally, 1978) which may be of concern. Another limitation concerns the fact that traditional personality factors were not included. Hence, future studies will have to determine the extent to which the associations established between constructs are being are accounted for by traditional personality factors such as neuroticisms and conscientiousness (cf., Stolarski and Matthews, 2016; Åström et al., 2019; Pyszkowska, 2020). Another potential concern is that we used a unidimensional measure of psychological flexibility, while researchers recently argued for a broader and multidimensional conceptualization of the construct (Rolffs et al., 2018). Of interest for future research will furthermore be to test the models presented in this study on a clinical sample, e.g., patients with depressision or anxiety that exhibit time perspective biases, to verify their generalizability. Given the results obtained, future studies regarding fusion and psychological rigidity in the context of Past Negative TP appears to an interesting direction in terms of possible clinical and therapeutic application. First, it may be of use in Accceptance and Commitment Therapy (Hayes et al., 1999), especially in a group of patients fused with their past memories, ruminations, or PTSD symptoms (Scarlet et al., 2016). Second, more generally, self-compassion and psychological flexibility are considered as learnable internal resources that can be enhanced via various therapeutic approaches (Neff, 2003), including classic cognitive-behavioral therapy (Lloyd et al., 2013; Diedrich et al., 2014) thus it would be applicable in enhancing one’s well-being and reducing the impact of negative automatic or core beliefs. From a clinical viewpoint, it is also worth noting that Zimbardo et al., 2012; Sword et al., 2014) developed a specific time perspective therapy aimed at reducing temporal biases (e.g., in PTSD patients). It is possible that such strategies could be effective to enhance psychological flexibility in a broader sense. Interventional research will have to evaluate these possibilities. In any case, time perspective biases are part of several forms of psychological suffering and should therefore be identified and considered as a target of intervention in clinical practice.

## Data Availability Statement

The raw data supporting the conclusions of this article will be made available by the authors, without undue reservation.

## Ethics Statement

The studies involving human participants were reviewed and approved by Ethics Committee, University of Silesia. The patients/participants provided their written informed consent to participate in this study.

## Author Contributions

AP designed the study, collected the data, and wrote a draft of the manuscript. MR performed parts of the analyses and made critical revisions of the manuscript. Both authors contributed to the article and approved the submitted version.

## Conflict of Interest

The authors declare that the research was conducted in the absence of any commercial or financial relationships that could be construed as a potential conflict of interest.

## References

[B1] Åström E. Rönnlund M. Adolfsson R. Carelli M. G. (2019). Depressive symptoms and time perspective in older adults: associations beyond personality and negative life events. *Aging Mental Health* 23 1674–1683. 10.1080/13607863.2018.1506743 30450950

[B2] Åström E. Seif A. Wiberg B. Carelli M. G. (2018). Getting “stuck” in the future or the Past: Relationships between Dimensions of Time Perspective, Executive Functions, and Repetitive Negative Thinking in Anxiety. *Psychopathology* 51 362–370. 10.1159/000494882 30522113

[B3] Beaumont E. Durkin M. Hollins Martin C. J. Carson J. (2016). Measuring relationships between self−compassion, compassion fatigue, burnout and well−being in student counsellors and student cognitive behavioural psychotherapists: a quantitative survey. *Couns. Psychother. Res.* 16 15–23. 10.1002/capr.12054

[B4] Bond F. W. Hayes S. C. Baer R. A. Carpenter K. M. Guenole N. Orcutt H. K. (2011). Preliminary Psychometric Properties of the Acceptance and Action Questionnaire–II: A Revised Measure of Psychological Inflexibility and Experiential Avoidance. *Behav. Therapy* 42 676–688. 10.1016/j.beth.2011.03.007 22035996

[B5] Carelli M. G. Wiberg B. M. Wiberg M. (2011). Development and construct validation of the Swedish Zimbardo Time Perspective Inventory. *Eur. J. Psychol. Assessm.* 24 220–227. 10.1027/1015-5759/a000076

[B6] Cernvall M. Skogseid E. Carlbring P. Ljungman L. Ljungman G. (2015). Experiential avoidance and rumination in parents of children on cancer treatment: Relationships with posttraumatic stress symptoms and symptoms of depression. *J. Clin. Psychol. Med. Settings* 23 67–76. 10.1007/s10880-015-9437-4 26462676

[B7] Costa J. Marôco J. Pinto-Gouvela J. A. Galhardo A. (2014). Validation of the psychometric properties of Acceptance and Action Questionnaire-II in clinical and nonclinical groups of Portuguese population. *Int. J. Psychol. Psychol. Therapy* 14 353–364.

[B8] Desmyter F. De Raedt R. (2012). The relationship between time perspective and subjective well-being of older adults. *Psychol. Belgica* 52 19–38. 10.5334/pb-52-1-19

[B9] Diedrich A. Grant M. Hofmann S. G. Hiller W. Berking M. (2014). Self-compassion as an emotion regulation strategy in major depressive disorder. *Behav. Res. Ther.* 58 43–51. 10.1016/j.brat.2014.05.006 24929927

[B10] Diener E. Emmons R. A. Larson R. J. Griffin S. (1985). The satisfaction with life scale. *J. Personal. Assessm.* 49 71–75. 10.1207/s15327752jpa4901_13 16367493

[B11] Doorley J. D. Goodman F. R. Kelso K. C. Kashdan T. B. (2020). Psychological flexibility: What we know, what we do not know, and what we think we know. *Soc. Personal Psychol. Compass.* 14:e12566. 10.1111/spc3.12566

[B12] Drake L. Duncan E. Sutherland F. (2008). Time perspective and correlates of well-being. *Time Soc.* 17 95–107.

[B13] Eriksson T. Germundsjö L. Åström E. Rönnlund M. (2018). Mindful self-compassion training reduces stress and burnout symptoms among practicing psychologists: a randomized controlled trial of a brief web-based intervention. *Front. Psychol.* 9:2340. 10.3389/fpsyg.2018.02340 30538656 PMC6277494

[B14] Ge J. Wu J. Li K. Zheng Y. (2019). Self-Compassion and Subjective Well-Being Mediate the Impact of Mindfulness on Balanced Time Perspective in Chinese College Students. *Front. Psychol.* 10 1–9. 10.3389/fpsyg.2019.00367 30853928 PMC6395405

[B15] Harris R. (2009). *ACT Made Simple: An Easy-to-Read Primer on Acceptance and Commitment Therapy.* Oakland, CA: New Harbinger Publications, Inc.

[B16] Hayes S. C. Strosahl K. Wilson K. G. (1999). *Acceptance and Commitment Therapy: An experiential approach to behavior change.* New York, NY: Guilford Press.

[B17] Hu L. T. Bentler P. M. (1999). Cutoff criteria for fit indexes in covariance structure analysis: Conventional criteria versus new alternatives. *Struct. Equat. Model. Multidiscipl. J.* 6 1–55. 10.1080/10705519909540118

[B18] Joeng J. R. Turner S. L. (2015). Mediators between self-criticism and depression: Fear of compassion, self-compassion, and importance to others. *J. Counsel. Psychol.* 62:453. 10.1037/cou0000071 25798874

[B19] Juczyński Z. (2001). *Narzêdzia pomiaru w promocji i psychologii zdrowia.* Warszawa: Pracownia Testów Psychologicznych PTP.

[B20] Kashdan T. B. Rottenberg J. (2010). Psychological flexibility as a fundamental aspect of health. *Clin. Psychol. Rev.* 30 865–878. 10.1016/j.cpr.2010.03.001 21151705 PMC2998793

[B21] Kleszcz B. Dudek J. E. Białaszek W. Ostaszewski P. Bond F. W. (2018). Właściwości psychometryczne polskiej wersji Kwestionariusza Akceptacji i Działania-II (AAQ-II). *Studia Psychol.* 56 1–20.

[B22] Lewin K. (1951) in *Field Theory in Social Science: Selected Theoretical Papers*, ed. Cartwright D. (New York, NY: Harper & Row).

[B23] Little T. D. Rhemtulla M. Gibson K. Schoemann A. M. (2013). Why the items versus parcels controversy needn’t be one. *Psychol. Methods* 18:285. 10.1037/a0033266 23834418 PMC3909043

[B24] Lloyd J. Bond F. W. Flaxman P. E. (2013). The value of psychological flexibility: Examining psychological mechanismns underpinning a cognitive burnout. *Work Stress* 27 181–199. 10.1080/02678373.2013.782157

[B25] Marshall E.-J. Brockman R. N. (2016). The Relationships between psychological flexibility, self-compassion, and emotional well-being. *J. Cognit. Psychother.* 30 60–72. 10.1891/0889-8391.30.1.60 32755906

[B26] Matsunaga M. (2008). Item parceling in structural equation modelling. *Primer Communicat. Methods Measur.* 2 260–293. 10.1080/19312450802458935

[B27] McAteer G. Gillanders D. (2019). Investigating the role of psychological flexibility, masculine self-esteem and stoicism as predictors of psychological distress and quality of life in men living with prostate cancer. *Eur. J. Cancer Care* 28:e13097. 10.1111/ecc.13097 31140670

[B28] McLean C. L. Fiorillo D. Follette V. M. (2018). Self-Compassion and Psychological Flexibility in a Treatment-Seeking Sample of Women Survivors of Interpersonal Violence. *Violence Victims* 33 472–485. 10.1891/0886-6708.v33.i3.472 30567859

[B29] Mead G. H. (1932). *The philosophy of the present.* Chicago, IL: Open Court.

[B30] Meyer E. C. Frankfurt S. B. Kimbrel N. A. DeBeer B. B. Gulliver S. B. Morrisette S. B. (2018). The influence of mindfulness, self-compassion, psychological flexibility, and posttraumatic stress disorder on disability and quality of life over time in war veterans. *J. Clin. Psychol.* 74 1272–1280. 10.1002/jclp.22596 29488629

[B31] Miyagawa Y. Taniguchi J. (2020). Self-Compassion and Time Perception of Past Negative Events. *Mindfulness* 11 746–755. 10.1007/s12671-019-01293-6

[B32] Muro A. Felin-Soler A. Castellà J. Devi J. Soler J. (2017). Does time perspective predict life satisfaction? A study including mindfulness as a measure of time experience in a sample of Catalan students. *Mindfulness* 8 655–663. 10.1007/s12671-016-0644-3

[B33] Neely M. E. Schallert D. L. Mohammed S. S. (2009). Self-kindness when facing stress: The role of self-compassion, goal regulation, and support in college students’ well-being. *Motivat. Emot.* 33 88–97. 10.1007/s11031-008-9119-8

[B34] Neff K. D. (2003). Self-Compassion: An Alternative Conceptualization of a Healthy Attitude Toward Oneself. *Self Ident.* 2 85–101. 10.1080/15298860309032

[B35] Neff K. D. Dahm K. A. (2015). “Self-compassion: What it is, what it does, and how it relates to mindfulness,” in *Handbook of mindfulness and Self-regulation*, eds Ostafin B. D. Robinson M. D. Meier B. P. (New York, NY: Springer), 121–137. 10.1007/978-1-4939-2263-5_10

[B36] Neff K. D. Germer C. K. (2013). A pilot study and randomized controlled trial of the mindful self-compassion program. *J. Clin. Psychol.* 69 28–44. 10.1002/jclp.21923 23070875

[B38] Nolen-Hoeksema S. Wisco B. E. Lyubomirsky S. (2008). Rethinking Rumination. *Perspect. Psychol. Sci.* 3 400–424. 10.1111/j.1745-6924.2008.00088.x 26158958

[B39] Nunnally J. C. (1978). *Psychometric theory*, 2nd Edn. New York, NY: McGraw-Hill.

[B40] Odou N. Brinker J. (2015). Self-compassion, a better alternative to rumination than distraction as a response to negative mood. *J. Posit. Psychol.* 10 447–457. 10.1080/17439760.2014.967800

[B41] Pandey R. Tiwari G. K. Parihar P. Rai P. K. (2021). Positive, not negative, self−compassion mediates the relationship between self−esteem and well−being. *Psychol. Psychother. Theory Res. Pract.* 94 1–15. 10.1111/papt.12259 31750614

[B42] Papastamatelou J. Unger A. Giotakos O. Athanasiadou F. (2015). Is time perspective a predictor of anxiety and perceived stress? Some preliminary results from Greece. *Psychol. Stud.* 60 468–477. 10.1007/s12646-015-0342-6

[B43] Philips W. J. (2018). Future-outlook mediates the association between self-compassion and wellbeing. *Personal. Individ. Differen.* 135 143–148. 10.1016/j.paid.2018.07.006

[B44] Pyszkowska A. (2020). Personality predictors of self-compassion, ego-resiliency and psychological flexibility in the context of quality of life. *Personal. Individ. Differen.* 161:109932. 10.1016/j.paid.2020.109932

[B45] Raes F. (2010). Rumination and worry as mediators of the relationship between self-compassion and depression and anxiety. *Personal. Individ. Differen.* 48 757–761. 10.1016/j.paid.2010.01.023

[B46] Raes F. Pommier E. Neff K. D. Van Gucht D. (2011). Construction and factorial validation of a short form of the Self-Compassion Scale. *Clin. Psychol. Psychother.* 18 250–255. 10.1002/cpp.702 21584907

[B47] Rolffs J. L. Rogge R. D. Wilson K. G. (2018). Disentangling components of flexibility via the hexaflex model: Development and validation of the Multidimensional Psychological Flexibility Inventory (MPFI). *Assessment* 25 458–482. 10.1177/1073191116645905 27152011

[B48] Rönnlund M. Åström E. Adolfsson R. Carelli M. G. (2018). Perceived stress in adults aged 65 to 90: Relations to facets of time perspective and COMT Val158met polymorphism. *Front. Psychol.* 9:378. 10.3389/fpsyg.2018.00378 29623060 PMC5874313

[B49] Rönnlund M. Åström E. Carelli M. G. (2017). Time Perspective in Late Adulthood: Aging Patterns in Past, Present and Future Dimensions, Deviations from Balance, and Associations with Subjective Well-Being. *Timing Time Percept.* 5 77–98. 10.1163/22134468-00002081

[B50] Rönnlund M. Koudriavtseva A. Germundsjö L. Eriksson T. Åström E. Carelli M. G. (2019). Mindfulness Promotes a More Balanced Time Perspective: Correlational and Intervention-Based Evidence. *Mindfulness* 10 1579–1591. 10.1007/s12671-019-01113-x

[B51] Samaie G. Farahani H. A. (2011). Self-compassion as a moderator of the relationship between rumination, self-reflection and stress. *Procedia Soc. Behav. Sci.* 30 978–982. 10.1016/j.sbspro.2011.10.190

[B52] Scarlet J. Lang A. J. Waiser R. D. (2016). “Acceptance and commitment therapy for posttraumatic stress disorder,” in *Complementary and Alternative Medicine for PTSD* eds Benedek D. M. Wynn G. H. (Oxford: Oxford University Press) 35–57.

[B53] Steenhaut P. Rossi G. Demeyer I. de Raedt R. (2018). How is personality related to well-being in older and younger adults? The role of psychological flexibility. *Int. Psychogeriatr.* 2018 1–14. 10.1017/S1041610218001904 30547852

[B54] Stoeber J. Lalova A. V. Lumley E. J. (2020). Perfectionism, (self-)compassion, and subjective well-being: A mediation model. *Personal. Individ. Differen.* 154:109708. 10.1016/j.paid.2019.109708

[B55] Stolarski M. Matthews G. (2016). Time perspectives predict mood states and satisfaction with life over and above personality. *Curr. Psychol.* 35 516–526. 10.1007/s12144-016-9515-2 27891043 PMC5104771

[B56] Stolarski M. Bitner J. Zimbardo P. G. (2011). Time perspective, emotional intelligence and discounting of delayed awards. *Time Soc.* 20 346–363. 10.1177/0961463x11414296

[B58] Stolarski M. Vowinckel J. Jankowski K. S. Zajenkowski M. (2016). Mind the balance, be contented: balanced time perspective mediates the relationship between mindfulness and life satisfaction. *Personal. Individ. Differen.* 93 27–31. 10.1016/j.paid.2015.09.039

[B60] Stolarski M. Zajenkowski M. Jankowski K. S. Szymaniak K. (2020). Deviation from the balanced time perspective: A systematic review of empirical relationships with psychological variables. *Personal. Individ. Differen.* 156:109772. 10.1016/j.paid.2019.109772

[B61] Straś-Romanowska M. (2005). Jakośæ życia w świetle załońeń psychologii zorientowanej na osobê. *Kolokwia Psychol.* 13 262–274.

[B62] Sword R. M. Sword R. K. M. Brunskill S. R. Zimbardo P. G. (2014). Time perspective therapy: A new time-based metaphor therapy for PTSD. *J. Loss Trauma* 19 197–201. 10.1080/15325024.2013.763632

[B63] Woodruff S. C. Glass C. R. Arnkoff D. B. Crowley K. J. Hindman R. K. Hirshhorn E. W. (2014). Comparing Self-Compassion, Mindfulness, and Psychological Inflexibility as Predictors of Psychological Health. *Mindfulness* 5 410–421. 10.1007/s12671-013-0195-9

[B64] Yang Y. Guo Z. Kou Y. Liu B. (2019). Linking self-compassion and prosocial behaviour in adolescents: The mediating roles of relatedness and trust. *Child Indicat. Res.* 12 2035–2049. 10.1007/s12187-019-9623-2

[B66] Zhang J. W. Howell R. Stolarski M. (2013). Comparing three methods to measure a balanced time perspective: The relationship between a balanced time perspective and subjective well-being. *J. Happin. Stud.* 14 169–184. 10.1007/s10902-012-9322-x

[B67] Zimbardo P. G. Boyd J. N. (1999). Putting time in perspective: A valid, reliable individual- difference metric. *J. Personal. Soc. Psychol.* 77 1271–1288. 10.1037/0022-3514.77.6.1271

[B68] Zimbardo P. G. Boyd J. N. (2008). *The Time Paradox – the new psychology of time that will change your life.* Mumbai:Free Press.

[B69] Zimbardo P. G. Sword R. M. Sword R. K. (2012). *The time cure: Overcoming PTSD with the new psychology of time perspective therapy.* San Francisco, CA:Jossey-Bass.

